# HIV/AIDS among youth in urban informal (slum) settlements in Kenya: What are the correlates of and motivations for HIV testing?

**DOI:** 10.1186/1471-2458-11-685

**Published:** 2011-09-03

**Authors:** Caroline W Kabiru, Donatien Beguy, Joanna Crichton, Eliya M Zulu

**Affiliations:** 1 African Population and Health Research Center (APHRC), APHRC Campus, Manga Close, Off Kirawa Road, P. O. Box 10787-00100, Nairobi, Kenya; 2 School of Social and Community Medicine, University of Bristol, Canynge Hall, 39 Whatley Road Bristol, BS8 2PS, UK; 3 African Institute for Development Policy (AFIDEP), Suite #29, 2nd Floor, Royal Offices, Mogotio Road (off Chiromo Lane) Westlands, P. O. Box 14688-00800, Nairobi, Kenya

## Abstract

**Background:**

Although HIV counseling and testing (HCT) is widely considered an integral component of HIV prevention and treatment strategies, few studies have examined HCT behavior among youth in sub-Saharan Africa-a group at substantial risk for HIV infection. In this paper we examine: the correlates of HIV testing, including whether associations differ based on the context under which a person gets tested; and the motivations for getting (or not getting) an HIV test.

**Methods:**

Drawing on data collected in 2007 from 4028 (51% male) youth (12-22 years) living in Korogocho and Viwandani slum settlements in Nairobi (Kenya), we explored the correlates of and motivations for HIV testing using the Health Belief Model (HBM) as a theoretical framework. Multinomial and binary logistic regression analyses were employed to examine correlates of HIV testing. Bivariate analyses were employed to assess reasons for or against testing.

**Results:**

Nineteen percent of males and 35% of females had been tested. Among tested youth, 74% of males and 43% of females had requested for their most recent HIV test while 7% of males and 32% of females reported that they were required to take their most recent HIV test (i.e., the test was mandatory). About 60% of females who had ever had sex received an HIV test because they were pregnant. We found modest support for the HBM in explaining variation in testing behavior. In particular, we found that perceived risk for HIV infection may drive HIV testing among youth. For example, about half of youth who had ever had sex but had never been tested reported that they had not been tested because they were not at risk.

**Conclusions:**

Targeted interventions to help young people correctly assess their level of risk and to increase awareness of the potential value of HIV testing may help enhance uptake of testing services. Given the relative success of Prevention of Mother-to-Child Transmission (PMTCT) services in increasing HIV testing rates among females, routine provider-initiated testing and counseling among all clients visiting medical facilities may provide an important avenue to increase HIV status awareness among the general population and especially among males.

## Background

HIV counseling and testing (HCT) is widely considered an integral component of HIV prevention and treatment strategies [1-4]. However, although recent estimates of the HIV/AIDS burden in Kenya underscore the particular vulnerability of youth to HIV, HIV testing and its determinants are largely understudied in this age group. In this study, we drew on data collected from young people living in Korogocho and Viwandani informal (slum) settlements in Nairobi, Kenya's capital city, to explore the linkages between sociodemographic characteristics, HIV-related psychosocial attributes, and HIV testing behavior.

### The HIV/AIDS Context in Kenya

As of 2007, 1.42 million Kenyans were living with HIV/AIDS, translating to an HIV prevalence of 7.1% [5]. Overall in Kenya, urban residents have a significantly higher risk of HIV infection (7.2%) than rural residents (6.0%) [6]. However, even in urban areas there are huge disparities in HIV prevalence with urban slum settlements having a significantly higher prevalence of HIV than non-slum urban areas. For example, a recent study conducted in two urban slum settlements in Nairobi [7] showed that the overall HIV prevalence in these slum settlements is estimated at 12%, which is much higher than the national average (7.1%) and the overall prevalence in Nairobi (7.0%) [6].

### HIV/AIDS among Kenyan Youth

Data from the 2007 Kenya AIDS Indicator Survey show a dramatic difference in HIV prevalence between 15-19 year olds (2.3%) and 20-24 year olds (5.2%) [5]. This difference suggests that many young people are infected during adolescence. Female youth are significantly more likely to be infected than their male peers [5]. For instance, among 15-19 years olds, 3.5% of females and 1% males are HIV positive, while among 20-24 year olds, 7.4% and 1.9%, respectively, are infected [5].

### HIV Counseling and Testing (HCT) in Kenya

HCT is an important component of government efforts to address HIV/AIDS in Kenya. Substantial efforts have been made by the Kenyan government and international development partners to increase access to voluntary counseling and testing (VCT) services in Kenya. Through these efforts, the number of VCT facilities, nationally, has increased significantly. For example, between 2000 and 2005, the number of testing facilities increased from 3 to over 500 [8]. Correspondingly, annual service uptake increased from 1000 to 380,000 people during this period [8]. Further, one of the objectives of the Government of Kenya's 2005-2015 Plan of Action under the Adolescent Reproductive Health Development Policy [9] is to establish and promote adolescent-friendly VCT services in order to improve access to and utilization of sustainable youth-friendly sexual and reproductive health services. Although the VCT to population ratio is highest for Nairobi (around one site for every 25,000 people) [8], few studies are available on access to HIV testing services in urban slum settlements in Nairobi, and sub-Saharan Africa, in general.

**Figure 1:**
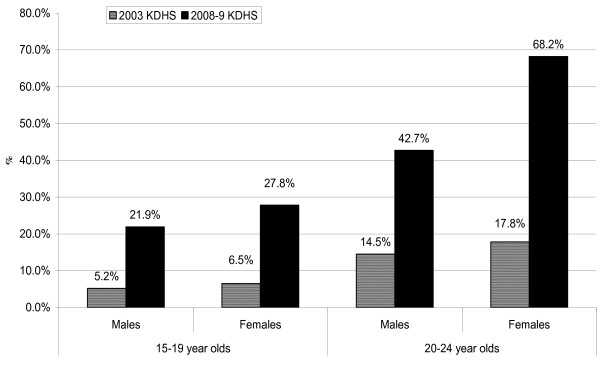
**Percentage of Kenyan youth who have been tested for HIV and received results**. The figure shows the percent distribution of youth who reported that they have ever been tested for HIV and received results (by sex and age) in the 2003 and 2008-09 Kenya Demographic and Health Surveys (KDHS) [6,10].

The 2008-9 Kenya Demographic and Health Survey (KDHS) [6] estimates show a substantial increase in the proportion of youth aged 15-24 years who have ever been tested for HIV and knew their HIV test results compared to 2003 KDHS [10] estimates. The proportions of youth reporting an HIV test during both surveys are illustrated in Figure 1. Overall, although these estimates reflect a significant improvement in the coverage of HIV counseling and testing, these numbers still fall short of the government's 2010 goal of 80% coverage [5].

### Correlates of HIV Testing among Youth

Policy and programmatic efforts to develop effective HIV prevention and treatment programs targeted towards youth living in resource-poor urban settings require empirical evidence on the drivers of HIV-related behavior including HIV testing and counseling. However, although there is substantial evidence that young people in urban slum settlements are more likely to engage in high risk sexual behavior and are at significantly higher risk for HIV infection than their peers in other settings [6,7,11-15], we know very little about the HIV testing behavior of youth in general. The limited existing research on the correlates of HIV testing among youth, in general, primarily focuses on attitudes toward HIV testing [16,17], intentions to test [18], differences in the timing of HIV testing from a life course perspective [19], or the correlates of HIV testing among convenience samples of specific groups of young people at high risk for infection such as gay and bisexual youth [20]. Existing studies provide useful insights on which youth are more likely to get tested for HIV. However, few examine the context under which a person gets tested-that is whether a person is offered the test, volunteers for a test, or whether the test is mandatory; and the motivation for the test. Yet, the motivation for testing and circumstances of the test might have significant bearing on subsequent behavior.

**Figure 2:**
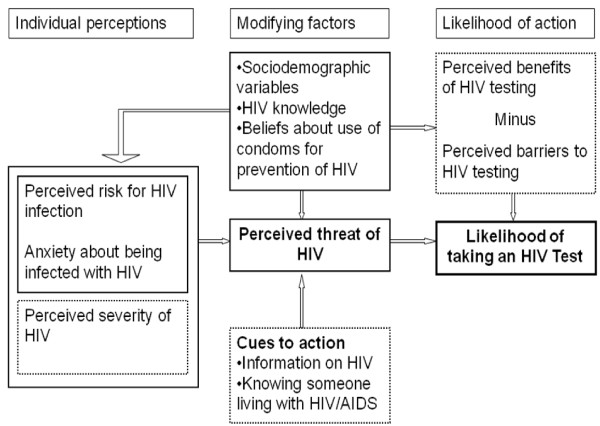
**The Health Belief Model: Application to HIV testing**. The figure illustrates the Health Belief Model (HBM) (modified from [21]). Variables that were not assessed in this study are shown in boxes with dashed lines.

The health belief model (HBM) model [21] provides a useful framework for studying HIV testing behavior among youth. The model posits that a person's likelihood of engaging in a particular health behavior is influenced by his or her perceived susceptibility to the health outcome (e.g., the subjective perception of the risk of contracting HIV), the perceived seriousness of the health outcome (e.g., one's feelings about the medical and social consequences of living with HIV/AIDS), the perceived benefits of preventive actions (e.g., using condoms or being tested for HIV), the perceived barriers or costs of taking a certain health action (e.g., stigma associated with being diagnosed with HIV), cues to action or cues that prompt one to take a certain action (e.g., media campaigns encouraging HIV testing); and self-efficacy or perceived competence in taking a particular health action to mitigate the health condition [21]. Sociodemographic and other structural variables may also influence these perceptions. For example, education may affect access to information about available services, such as HIV testing. Figure 2 illustrates the HBM and shows how study variables fit into the model.

In this study, we 1) explored the linkages between young people's sociodemographic characteristics, HIV-related psychosocial attributes, and HIV testing behavior; and 2) investigated the reasons young people living in urban slum settlements in Nairobi give for testing (or not testing) for HIV. With respect to the first aim, we specifically investigated differences between young people based on 1) HIV testing status (never versus ever tested), and 2) among young people who requested for an HIV test -- which we considered a voluntary test --, those who were offered the test, and those required to take a test (e.g., for employment purposes) -- which we considered a mandatory test. With respect to the HBM constructs, we hypothesized that youth who believed that they were more susceptible to HIV infection, who were worried about HIV infection, who had greater HIV knowledge, and who believed in the benefits of the preventive action of condom use were more likely to have been tested and to have requested an HIV test as opposed to being offered the test or being required to take a test.

## Methods

### Study Setting

We used data collected from youth in two slum settlements in Nairobi. Korogocho slum settlement is one of the oldest and most congested settlements within the city, and many residents have lived there for many years. Viwandani slum settlement is situated in Nairobi's industrial area, and attracts a youthful, highly mobile migrant population seeking employment in industry. Although the slum settlements have different demographic and livelihood profiles, both are characterized by inadequate access to formal health, education and other social services [22,23]. The combined effects of inadequate health services, poverty, and difficult socio-environmental conditions increase slum dwellers' vulnerability to poor health outcomes [24,25].

Previous studies suggest that urban poverty [12,24] and the high mobility of the urban poor [26,27] may increase vulnerability to HIV/AIDS. According to Krishnan [28], poverty may increase vulnerability to HIV through several pathways including: a greater likelihood to engage in transactional sex; limited access to formal education and skills, which heightens economic dependency especially for women and girls; and limited access to HIV preventive services and health information. On the other hand, high mobility may increase vulnerability to HIV through increased access to casual sexual partners and weakened social controls [27]. Studies conducted in Kenya show that slum dwellers fare much worse in terms of risky sexual behavior when compared to their wealthier urban counterparts or those living in rural areas [12,14,29]. For example, Zulu and colleagues [14] observed a 5 year difference in the median age at first sex between those who grew up in slum settlements and those who grew up in other parts of the city. This difference in age at sexual debut remained significant even after controlling for sociodemographic variables such as religion and schooling. Increased vulnerability to HIV among youth living in these resource-poor settings is reflected in the relatively high HIV prevalence observed in 2008; 6% and 3% among 15-19 year old females and males respectively, and 9% and 3% among 20-24 year old females and males, respectively [7]. The high HIV prevalence underscores the need to understand the drivers of HIV-related behavior in order to inform HIV prevention programs targeted towards slum residents.

### Study Design, Participants and Procedures

We drew on baseline data from the 3-year (2007-2010) prospective Transition-To-Adulthood (TTA) study. The TTA's general objective was to identify protective and risk factors in the lives of youth growing up in the two slum settlements, and to examine how these factors influence their transition to adulthood. The study was nested in the Nairobi Urban Health and Demographic Surveillance System (NUHDSS), which covers nearly 72,000 people in 28,000 households every year. For the TTA project, a random sample of youth was selected from 2007 NUHDSS household records. During the first wave of data collection (October 2007 to June 2008), 4058 youth aged 12-22 years were interviewed. This number reflects a 77% response rate among eligible randomly-selected youth aged 12-22 years who were resident in the NUHDSS (N = 5,281). Less than 5% of youth who were approached to participate in the study refused to be interviewed. The relatively low response rate is therefore primarily attributable to difficulties in locating some youth, given the high mobility of residents in the area [26]. Respondents did not differ from non-respondents by sex; however, respondents were younger (*p <*.10) and more likely to be from Korogocho (*p <*.05). In this study, we limit our analyses to the 4028 (99% of respondents) who answered questions on HIV testing.

A comprehensive interview questionnaire was administered that included questions covering sociodemographic characteristics (e.g. school and religious affiliation); key health and other concerns; and sexual behavior. The questionnaire also included a module on HIV knowledge, HIV testing experiences, and reasons for getting (or not getting) tested. The interviews were conducted in Kiswahili by male and female interviewers, many of whom had previous experience working in the two slum settlements. Most of the interviewers were younger than 30 years of age. Interviewers underwent a 5-day training program that included, among other topics: an introduction to the project; the roles of interviewers, research ethics; and familiarization with the questionnaire. The training also included mock interviews with other interviewers, as well as pilot interviews with a group of youth living in the two slum settlements but outside the study area.

Ethical approval was granted by the Kenya Medical Research Institute's ethical review board. Verbal or signed informed consent was obtained from all respondents. For respondents aged 12-17, consent was also requested from the parent or guardian.

### Measures

#### Outcome variables

The primary outcome variables measured HIV testing status. The first variable, HIV testing status, was constructed based on the response to the question: *I don't want to know the results, but have you ever been tested to see if you have the AIDS virus? *The response categories for this variable were: Yes (coded "1") and No (0). The second variable, the context under which the recent HIV test was taken, was derived from the response to the following question that was asked to youth who had ever been tested: *The last time you had the test, did you yourself ask for the test, was it offered to you and you accepted, or was it required? *The response categories for this variable were: respondent asked for test (coded "1"), respondent was offered the test and accepted (2), and respondent was required to take the test (3). We note here, that testing under prevention of mother-to-child transmission (PMTCT) services is widespread and the interpretation of testing provided under PMTCT programs as 'required' versus 'offered' may be based on women's perceptions rather than a difference in their treatment by service providers.

All youth who had ever been tested were asked to provide reasons for their most recent HIV test. Youth who had never been tested were asked why they had never been tested. To understand the testing process, we also examined where young people got tested for HIV, whether counseling was offered and whether respondents received their test results.

#### Independent variables

Drawing from the HBM, the primary independent variables were HIV/AIDS knowledge, perceived susceptibility to HIV, level of anxiety about getting infected with HIV and attitudes towards condom use for HIV prevention. HIV/AIDS knowledge was assessed using a 13-item scale derived from existing instruments including the KDHS [10] (sample question: *In your opinion, can a person get HIV/AIDS from being bitten by mosquitoes or other insects?*). Cronbach's alpha was used to assess internal consistency of scores, a measure of the extent to which items that measure the same construct are correlated [30,31]. The internal consistency of the HIV/AIDS knowledge scale (0.59) was low but within the acceptable range [32,33]. We therefore created a composite HIV/AIDS knowledge index using standardized (mean equal to zero and standard deviation equal to one) values of individual items all scored in the positive direction. Perceived susceptibility to HIV/AIDS was measured using a single question: *What are your chances of not getting HIV/AIDS? *Possible responses were high, about 50-50, and low. Anxiety about getting infected with HIV was measured using a single question: *How worried are you about getting HIV/AIDS? *Possible responses were: very worried, somewhat worried, or not worried. Attitudes towards condom use for HIV prevention were assessed using a single item: *It's a good idea to use condoms to protect against getting AIDS*. Possible responses were: agree, disagree, or don't know.

We also considered sociodemographic characteristics including respondent's sex, age, schooling status at the time of interview, study site, and religious affiliation. Previous research shows that a history of pregnancy is associated with HIV testing [34,35]. Thus, we included whether the respondent had ever been or made someone pregnant in the models.

### Analyses

To explore the linkages between young people's sociodemographic characteristics, HIV-related psychosocial attributes, and HIV testing behavior, we first used logistic regression models to compare youth who had been tested for HIV to those who had never been tested. We then used multinomial logit regression models to examine differences among female respondents who requested their most recent HIV test, those offered the test, and those required to take a test. Given the small number of male respondents who reported that their most recent test was required (n = 27), we created a single category coded "0" for those offered the test and those required to take the test. We then ran a logistic regression model to compare the combined group of those offered the test and those required to take the test to those who requested an HIV test. We examined differences across outcome groups based on the primary explanatory variables derived from the HBM as well as sociodemographic and behavioral variables that may influence HIV testing. In sub-Saharan Africa, HIV is predominantly transmitted via sexual intercourse, and previous sexual behavior may influence perceived need for HIV testing. We therefore included sexual experience (ever--coded 1--versus never having had sex--coded 0) as an independent variable.

Reasons for testing (or not testing) may also differ based on whether or not a person has ever had sexual intercourse. Thus, to investigate the reasons young people living in the study communities give for testing decisions, we ran cross tabulations of reasons given by sexual experience. All analyses were conducted separately for males and females as we assume that factors associated with HIV testing behavior are gender sensitive.

## Results

### Characteristics of Study Population

Descriptive characteristics of the sample are summarized in Table 1. Fifty-one percent of respondents were males. Three quarters of respondents self-identified as Christians. Sixty-one percent of males and 53% of females were in school at the time of the survey. Eighteen percent of females were married compared to 4% of males. Twenty-two percent of females had ever been pregnant compared to 4% of males who had ever made someone pregnant. Thirty-four percent of respondents were sexually experienced. Almost twice as many females (35%) as males (19%) had been tested for HIV. Males and females did not differ on perceived chances of getting HIV, anxiety about being infected with HIV, HIV knowledge, and attitudes towards condom use for HIV prevention.

**Table 1:** Descriptive characteristics of respondents, by gender

	Male	Female	Total
		
	N = 2,037	N = 1,991	N = 4,028
*Sociodemographics*			
Mean age in years (standard deviation)	16.6	16.8	16.7
	(3.01)	(2.99)	(3.00)
Study site (%)			
Korogocho	49.0	48.5	48.8
Viwandani	51.0	51.5	51.2
Religion (%)			
Catholics	27.5	27.7	27.6
Protestants	21.9	22.7	22.3
Pentecostal or other Christians	22.4	28.0	25.2
Muslims	12.4	11.2	11.8
Other	15.8	10.5	13.2
% In school	60.7	52.9	52.9
% Ever married	3.9	18.4	11.1
% Ever pregnant/made someone pregnant	4.4	21.7	13.0
*Behavioral characteristics and HIV-related variables*			
% Ever had sex	31.9	35.5	33.7
% Ever tested for HIV	19.3	34.8	27.0
Perceived chances of getting HIV			
High	3.1	3.4	3.2
About 50-50	36.0	36.1	36.1
Low	60.9	60.5	60.7
Anxiety about being infected with HIV			
Very worried	34.1	34.2	34.2
Somewhat worried	15.3	15.4	15.4
Not worried	50.6	50.4	50.5
Mean HIV knowledge (standard deviation)	10.0	10.0	10.0
	(0.40)	(0.42)	(0.41)
It is a good idea to use condoms to prevent HIV infection (%)			
Agree	85.8	82.5	84.2
Disagree	11.1	13.1	12.1
Don't know	3.1	4.4	3.7

### Experiences with Testing

About half of the males received their most recent HIV test in a VCT center (Table 2). As expected, the proportions differ based on the reason for testing: 60% of male respondents who requested for an HIV test got tested in a VCT center compared to 29% and 7% of those offered the test and those required to take the test respectively. Less than a third (29%) of females received their most recent HIV test at a VCT center. As with males, a greater proportion of females who requested for an HIV test (53%) got tested in a VCT center compared to those who were offered the test (16%) and those who were required to take the test (8%). Among women who were required to take the test, close to three-quarters (73%) received their HIV test in a government clinic or hospital.

**Table 2:** Percentage distribution of young people who have been tested for HIV, by place of last test, receipt of counseling, and whether respondent obtained test results, according to sex and reason for testing

	Male				Female			
		
	Asked for test	Was offered the test and accepted	Required to take the test	Total	Asked for test	Was offered the test and accepted	Required to take the test	Total
Place where last HIV test was obtained								
Government clinic/hospital	20.6	12.3	40.7	20.5	27.9	46.6	72.8	46.9
Private clinic or hospital	10.7	5.5	3.7	9.2	12.6	6.3	16.6	12.2
Non-governmental clinic	0.0	8.2	11.1	2.3	1.0	3.4	0.5	1.5
Mobile clinic	7.2	38.4	29.6	14.6	5.4	22.7	1.8	8.7
VCT center	59.8	28.8	7.4	50.4	52.7	15.9	7.8	29.1
Other	1.7	6.9	7.4	3.1	0.3	5.1	0.5	1.6
Was counseling offered at last HIV test								
Yes	96.9	94.4	85.2	95.6	96.2	91.4	87.1	92.1
No	3.1	5.6	14.8	4.4	3.8	8.6	12.9	7.9
Did respondent obtain last test results								
Yes	98.3	78.1	92.6	94.1	97.6	85.8	97.3	94.5
No	1.7	21.9	7.4	5.9	2.4	14.2	2.8	5.5
N	291	73	27	391	294	176	218	688

Overall, although the vast majority of respondents (over 90%) received counseling during their most recent HIV test, about 15% and 13% of males and females, respectively, who were required to take an HIV test did not receive counseling. Over 90% of respondents received their HIV test results. However, 22% of males and 14% of females who were offered the test did not receive their test results. Subsequent analyses (results not shown) indicate that respondents who received counseling were more likely to receive their test results.

### Correlates of having been Tested for HIV

To explore the correlates of having been tested for HIV, we first compared young people who had ever been tested for HIV to those reporting no previous test. At the bivariate level (results not shown), males with high HIV knowledge, who were somewhat worried about being infected with HIV relative to those who were not worried, who were older, who were living in Viwandani, were Catholic relative to being Muslim, were out of school, were married, had made someone pregnant, and who had engaged in sexual intercourse were more likely to have ever been tested for HIV. Among females, those with high HIV knowledge, those who believed that condoms prevent HIV transmission, those perceiving a 50-50 chance of HIV infection relative to low perceived risk, those who were somewhat worried about being infected with HIV relative to not being worried, who were older, were Catholic relative to being Muslim, were out of school, were married, who had ever been pregnant, and who had ever had sexual intercourse were more likely to have ever been tested for HIV.

In the adjusted models among males (Table 3), only age, area of residence and sexual experience were associated with HIV testing at the .05 level of significance. Specifically, older males, males living in Viwandani, and sexually experienced males were more likely to have ever been tested for HIV. Among females, age, religion, pregnancy, and sexual experience were associated with HIV testing at the .05 level of significance. Because of the strong association between pregnancy and HIV testing among females, we ran an additional model (results not shown) excluding the pregnancy variable. Once we dropped pregnancy from the model, marital and schooling status became statistically significant with married females and youth who were out of school being more likely to have ever been tested.

**Table 3:** Estimated odds ratios (and confidence intervals) from logistic regression analyses of HIV testing status, by sex

	Males		Females	
	Odds Ratios	[95% CI]	Odds Ratios	[95% CI]
HIV knowledge	1.39	[0.89,2.18]	0.92	[0.68,1.25]
It is a good idea to use condoms to prevent HIV infection (ref. agree)				
Disagree	1.28	[0.86,1.91]	1.1	[0.74,1.62]
Don't know	1.27	[0.54,3.00]	0.49*	[0.23,1.03]
Perceived chances of getting HIV (ref. low)				
About 50-50	1.14	[0.86,1.52]	0.85	[0.64,1.13]
High	0.76	[0.34,1.72]	1.52	[0.79,2.94]
Anxiety about being infected with HIV (ref. not worried)				
Very worried	0.99	[0.74,1.31]	1.12	[0.83,1.51]
Somewhat worried	0.98	[0.68,1.42]	1.18	[0.80,1.75]
Age	1.28***	[1.20,1.35]	1.27***	[1.20,1.35]
Viwandani (ref. Korogocho)	1.48***	[1.14,1.93]	1.07	[0.82,1.40]
Religion (ref. Catholic)				
Protestants	1.37*	[0.98,1.91]	0.98	[0.69,1.40]
Pentecostal or other Christians	0.97	[0.68,1.39]	1.03	[0.74,1.42]
Muslims	0.78	[0.49,1.25]	0.37***	[0.21,0.65]
Other	0.73	[0.50,1.08]	1.09	[0.69,1.72]
In school (ref. out of school)	0.88	[0.65,1.20]	0.78	[0.55,1.10]
Married (ref. not married)	1.37	[0.71,2.63]	1.1	[0.69,1.75]
Ever pregnant (ref. never pregnant)	1.18	[0.63,2.19]	7.29***	[4.61,11.53]
Ever sex (ref. never had sex)	1.86***	[1.38,2.52]	2.37***	[1.67,3.36]
				
Wald chi-square	261.48***		542.8***	
Pseudo R^2^	0.1497		0.3771	
N	2015		1971	

CI = Confidence interval; ref = Reference category

*p < 0.10, **p < 0.05, ***p < 0.01

### Correlates of the Context under which the Most Recent HIV Test was taken

To examine the net association between the independent variables and the context under which the most recent HIV test was taken among those who had ever been tested, we ran a multinomial logit model (for females) and binary logistic model (for males). Table 4 presents the adjusted relative risk ratios (multinomial model) and odds ratios (binary logistic model) for variables included in the models. HIV/AIDS knowledge was not associated with the context under which the most recent HIV test was taken among males and females. Males who did not know whether it was a good idea to use condoms to prevent HIV infection were 5.7 times more likely to have been offered or required to take the test than to have requested the test compared to those who agreed with the statement. Females who responded that they 'did not know' to the question regarding condoms were more likely to have been required to take the test. Among females, relative to those who perceived that their chances of getting HIV were low, females who perceived a 50-50 chance of infection were marginally less likely to have had a mandatory than voluntary test. Thus, perceiving that one is at risk for infection may be associated with greater VCT use. Females who were 'somewhat' worried about being infected with HIV were more likely to have been offered the test than to have requested the test.

**Table 4:** Logistic (males) and multinomial logistic (females) regression on predictors of the context under which the recent HIV test was taken, by gender

	Males		Females			
		
	Offered/required vs requested		Offered vs. requested		Required vs. requested	
	Odds Ratios	[95% CI]	RRR	[95% CI]	RRR	[95% CI]
HIV knowledge	0.60	[0.28,1.28]	0.90	[0.48,1.70]	0.84	[0.43,1.66]
It is a good idea to use condoms to prevent HIV infection (ref. agree)						
Disagree	1.71	[0.84,3.48]	0.74	[0.35,1.57]	1.43	[0.67,3.04]
Don't know	5.68**	[1.18,27.39]	1.47	[0.13,16.98]	10.49**	[1.29,85.09]
Perceived chances of getting HIV (ref. low)						
About 50-50	1.52	[0.86,2.69]	1.39	[0.89,2.18]	0.64*	[0.38,1.07]
High	1.03	[0.16,6.62]	0.43	[0.11,1.71]	0.76	[0.19,2.98]
Anxiety about being infected with HIV (ref. not worried)						
Very worried	0.81	[0.44,1.49]	0.78	[0.48,1.28]	0.92	[0.56,1.51]
Somewhat worried	1.73	[0.85,3.53]	2.20***	[1.27,3.83]	1.2	[0.60,2.41]
Age	0.83***	[0.73,0.95]	0.96	[0.86,1.08]	0.88*	[0.76,1.01]
Viwandani (ref. Korogocho)	0.78	[0.45,1.35]	1.10	[0.71,1.68]	0.51***	[0.32,0.80]
Religion (ref. Catholic)						
Protestants	1.59	[0.82,3.09]	1.03	[0.60,1.77]	1.37	[0.77,2.46]
Pentecostal or other Christians	1.31	[0.63,2.74]	0.75	[0.41,1.35]	2.07**	[1.13,3.80]
Muslims	1.95	[0.78,4.89]	1.33	[0.53,3.35]	1.30	[0.48,3.52]
Other	0.49	[0.19,1.24]	0.92	[0.52,1.63]	0.68	[0.33,1.40]
In school (ref. out of school)	1.29	[0.70,2.39]	2.14**	[1.11,4.14]	0.64	[0.24,1.69]
Married (ref. not married)	0.23*	[0.04,1.19]	0.84	[0.48,1.48]	0.94	[0.53,1.66]
Ever pregnant (ref. never pregnant)	2.54	[0.70,9.28]	4.00***	[2.18,7.35]	36.54***	[13.17,101.34]
Ever sex (ref. never had sex)	0.95	[0.51,1.78]	0.82	[0.43,1.57]	0.48	[0.16,1.42]
Wald chi-square	46.33***		176.25***			
Pseudo R2	0.1062		0.1921			
N	382		677			

RRR = Relative Risk Ratio; CI = Confidence Interval; ref = Reference category

*p < 0.10, **p < 0.05, ***p < 0.01

Among males, age was the only sociodemographic variable associated with the context under which the most recent test was taken at the .05 level of significance. Older males were less likely to have been required/offered the test than to have requested the test. Among females, on the other hand, area of residence, religion, schooling status, and pregnancy history were associated with the reason for testing. Females who were in school were more likely to have been offered the test than to have requested the test. Females who had ever been pregnant were close to 36.5 times more likely to have been required to take the test and 4.0 times more likely to have been offered the test compared to those who had never been pregnant. As with the analyses on the correlates of having tested for HIV, we examined the effect of dropping pregnancy in the multinomial regression model. Once pregnancy was dropped from the model based on data from females (results not shown), schooling, marital status and sexual experience became strongly associated with the outcome variable. Specifically, females in school were more likely to have requested their most recent HIV test than to have been required to take the test, while married respondents and those who had ever had sex were more likely to have been required to take an HIV test than to have requested the test.

### Motivations For or Against Testing

To further understand the motivations for or against testing, we asked respondents to give the primary reason why they had or had not been tested for HIV. A summary of the feedback received from respondents is presented by sexual experience and sex in Table 5. Forty percent of males and 43% of females who had never had sex had not been tested mainly because they were not sexually active. Among those who had ever had sex, 37% of males and 44% of females reported that they had not been tested because they were not at risk. Close to a fifth of sexually experienced respondents had not been tested because they did not want to know their status. Among males who had been tested, the predominant reason for getting tested was concern about own status. Ten percent of males who had ever been tested and never had sex had been encouraged to get tested by a counselor, peer educator, parents, or peers. For females, on the other hand, close to 75% of those who had been tested and never had sex got tested because they wanted to know their status compared to 34% of those who were sexually experienced. Further analysis showed that 61% females who had been tested and never had sex requested their most recent HIV test compared to 36% of their sexually experienced counterparts (results not shown). About 60% of females who had ever had sex received an HIV test because they were pregnant or because testing was part of pre-natal care.

**Table 5:** Reasons for testing (among ever tested) or not testing (among never tested), by gender and sexual experience

	Male			Female		
		
	Never had sex	Ever had Sex	Total	Never had sex	Ever had Sex	Total
*Reason for not having been tested*						
Not sexually active	39.9	5.3	31.1	42.7	1.0	36.4
Not at risk for other reasons	28.0	36.6	30.2	29.6	44.4	31.8
Don't know where to go	4.7	1.7	4.0	4.3	3.6	4.2
Costs too much/lack of money	0.2	0.2	0.2	0.4	0.5	0.4
Can get infection from test	0.2	1.2	0.5	0.7	0.5	0.7
Don't want to know status	4.7	18.1	8.1	3.6	17.4	5.7
Someone might see me	0.7	1.7	0.9	0.4	1.0	0.5
Trust myself or partner/sure of status/know myself	3.8	9.6	5.3	3.0	6.1	3.5
Has not thought about it	2.3	3.9	2.7	1.7	6.1	2.4
No reason/nothing	2.9	5.5	3.6	4.4	4.6	4.4
Lacks time/too busy	2.0	4.1	2.6	1.2	3.6	1.5
Fear/Afraid to know status	0.7	2.7	1.2	0.5	2.6	0.9
Still in school/too young	2.4	0.5	1.9	1.2	0.5	1.1
Not interested/don't feel like it	0.4	1.0	0.6	0.3	3.1	0.7
Other/missing responses	7.0	8.0	7.2	6.1	5.1	5.9
N	1,227	415	1,642	1,101	196	1,297
*Reason for being tested*						
To know status	80.9	89.3	85.9	75.3	34.1	44.9
Pregnant or part of pre-natal	--	0.4	0.3	--	59.4	44.2
I'm sexually active	--	0.0	0.3	--	0.6	0.6
Encouraged by counselor	1.9	0.9	1.3	4.4	1.6	2.3
Encouraged by peer educator	3.8	0.4	1.8	3.3	0.4	1.2
Encouraged by parent	1.3	0.4	0.8	1.7	0.0	0.4
Encouraged by peer	2.6	1.3	1.8	1.1	0.6	0.7
To get married	0.6	0.4	0.5	0.0	0.0	0.0
Partner told me to do so	0.6	0.9	0.8	1.1	1.2	1.2
Concern about a partner	0.0	0.4	0.3	0.0	0.4	0.3
Required for job/school	1.3	1.3	1.3	2.2	0.4	0.9
Blood Donation	1.9	0.9	1.3	0.6	0.2	0.3
Was Sick	1.9	0.4	1.0	3.3	0.4	1.2
Other/missing responses	3.2	3.0	2.8	5.0	0.8	1.9
N	157	233	390	182	510	692

## Discussion

The high prevalence of HIV/AIDS among young people in sub-Saharan Africa [36] has stimulated research and programmatic efforts to understand and address their sexual and reproductive health. In this study, we drew on data collected in two urban slum settlements in Nairobi City to 1) explore the linkages between young people's sociodemographic characteristics, HIV-related psychosocial attributes, and HIV testing behavior; and 2) investigate the reasons young people give for getting (or not getting) tested for HIV.

The bivariate analyses revealed the theoretically-expected association between HIV testing and the HBM-based constructs of HIV/AIDS knowledge, perceived susceptibility to HIV, level of anxiety about getting infected with HIV and attitudes towards condom use for HIV prevention. However, in the multivariable models only attitudes towards condom use was significantly associated with HIV testing among females. We note that we used a less exhaustive set of measures since this study was not designed as a test for HBM. Nonetheless, some of our findings do lend some support for the model. For example, at the bivariate level, we observed that, for females, youth who perceived that they were at some risk for HIV were more likely to have ever been tested. This echoes the voices of tested youth in the two slum settlements, majority of whom stated that they had been tested because they were concerned about their HIV status. Denison and colleagues [18] in their study of youth aged 16-19 years in Ndola, Zambia also found that not wanting to be worried and wanting to know one's status were frequent reasons for willingness to be tested for HIV.

Of concern, however, is that among young people who had never been tested and were sexually experienced, close to two-fifths stated that they had not been tested because they were not at risk. Similar results were noted in a study conducted by Merchan-Hamann and colleagues [37] in Brazil where the most common reason given by sexually experienced adolescents for not testing was that they were not at risk for HIV or that they trusted their sexual partner. Given that condom use among young people in Nairobi is relatively low even in instances where young people have multiple sexual partnerships [6], being sexually experienced presents high risk for HIV infection. These results suggest that the educational campaign aimed at getting young people to understand that having unprotected sex is a risk factor for HIV infection, irrespective of what partners one is involved with, is not getting through to many young people. Thus, programmatic efforts to enable young people to accurately assess their levels of risk based on prior behavior may lead to increased use of HCT services. However these findings also underscore the need for alternative approaches beyond VCT, which "typically serve the 'worried well'" [38] (p. 861), such as routine provider-initiated testing.

We observed significant gender differences in factors associated with HIV testing among youth in slum settlements. In particular, we found that young females are more likely to be required or offered an HIV test than are males, and that a substantial proportion of females who reported an HIV test were tested because they were pregnant. These findings are not surprising, given the widespread promotion of PMTCT interventions in the region [39]. Previous studies among Kenyan [19] and South African [40] youth have also shown that HIV testing among females is highly associated with pregnancy status. Targeting pregnant youth for HIV testing is important in preventing pediatric HIV/AIDS and provides a potentially important avenue to reach partners of pregnant youth. Farquhar and colleagues' [41] study on antenatal couple counseling in Kenya highlights potential benefits of partner participation in adoption of preventive strategies and women's uptake of PMTCT services. In this respect, the Kenya Ministry of Health's guidelines for PMTCT services in Kenya [42] emphasize the need for partner involvement in PMTCT services. Nevertheless, it is important that HIV testing interventions target both males and females who may not be reached through PMTCT initiatives [35]. As noted by MacPhail and colleagues [40] in their study among youth in South Africa, a large proportion of young people visit health care facilities for various health services. Thus, drawing on the relative success of PMTCT services in increasing HIV testing rates among pregnant female youth, routine provider-initiated testing and counseling among all clients visiting medical facilities may provide an important avenue to increase HIV status awareness particularly for young men and non-pregnant women. Previous studies have shown the feasibility of provider-initiated HIV testing programs in limited resource-settings [43,44].

Study findings suggest that there are substantial differences in HIV testing rates between the two slum settlements with males in Viwandani being more likely to have ever been tested than their peers in Korogocho and females in Viwandani being more likely than females in Korogocho to have requested an HIV test relative to being required to have one. Preliminary unpublished data from a recent study conducted in these two slum settlements [45] shows that 41% of the 160 clinics and health centers in Korogocho surveyed in 2008 provided HCT services. In Viwandani, 34% of the 134 clinics offered HCT services. However, the same data showed that a greater proportion of health facilities in Viwandani (56%) than Korogocho (34%) had staff qualified to provide HCT services. These data suggest that in practice, residents of Viwandani may have a relative advantage in accessing HIV testing services. Given the high prevalence of HIV/AIDS in these urban slum settlements, it is important to ensure that residents have adequate access to HIV prevention and treatment services. Further, we note that although one might expect that majority of youth who request an HIV test would be tested in VCT centers, which are often low cost, we found that substantial proportions of youth who requested an HIV test received their test in other health care facilities. Data from the 2007 Kenya AIDS Indicator Survey [5] show that only about one-fifth of Kenyan adults get tested in VCT centers or mobile units. We posit that some people may prefer to be tested at a general health center because this offers more privacy. Thus, ensuring that health care facilities in urban slum settlements are equipped with HCT services may increase access to testing services.

Finally, the guidelines issued by the Kenyan Ministry of Health recommend that appropriate counseling should follow all testing [46]. Yet, we observed that counseling is not enforced universally. Given that those who received counseling were more likely to receive their test results, it is important to take steps to ensure compliance with government policies and guidelines on HIV testing.

Our study findings should be interpreted in light of several limitations. First, the study relied on self-reported data that are subject to response bias. Second, the cross-sectional nature of the study does not allow us to make causal inferences. Finally, the measures of the reasons for being tested or not being tested involved only the main reason in each case. Since individual decision-making surrounding HIV testing is likely to involve many different factors, this may under-represent some important secondary factors that affect health behavior.

## Conclusions

The strong associations between HIV testing and pregnancy status among females and between HIV testing status and perceived risk have implications for policy and programmatic efforts aimed at increasing HIV testing among young people. Specifically, the higher level of testing among females compared to males during pre-natal care suggests that routine testing may be a viable option for increasing testing coverage among males as well. In addition, the finding that the decision to test may not always be driven by one's level of sexual risk-taking underscores the need for programs that focus on enabling young people to accurately assess their levels of risk. In particular, programs should help young people understand that having unprotected sex puts them at risk, irrespective of the partner they have sex with.

## Competing interests

The authors declare that they have no competing interests.

## Authors' contributions

CWK conceptualized the manuscript idea, conducted the data analyses, participated in the literature review, and prepared the first draft of the manuscript. DB made substantive contributions to the conceptualization of the manuscript and supported data analyses. JC made substantive contributions to the conceptualization of the study and assisted with revising the manuscript. EZ was the primary investigator for the larger study from which the paper is drawn, made substantive contribution to the conceptualization of the manuscript, and assisted with revising the manuscript. All authors critically reviewed the manuscript. All authors have read and approved the final manuscript.

## Pre-publication history

The pre-publication history for this paper can be accessed here:

http://www.biomedcentral.com/1471-2458/11/685/prepub
